# Characterization of Supragingival Plaque and Oral Swab Microbiomes in Children With Severe Early Childhood Caries

**DOI:** 10.3389/fmicb.2021.683685

**Published:** 2021-06-25

**Authors:** Vivianne Cruz de Jesus, Mohd Wasif Khan, Betty-Anne Mittermuller, Kangmin Duan, Pingzhao Hu, Robert J. Schroth, Prashen Chelikani

**Affiliations:** ^1^Manitoba Chemosensory Biology Research Group, Department of Oral Biology, University of Manitoba, Winnipeg, MB, Canada; ^2^Children’s Hospital Research Institute of Manitoba (CHRIM), Winnipeg, MB, Canada; ^3^Department of Biochemistry and Medical Genetics, University of Manitoba, Winnipeg, MB, Canada; ^4^Department of Preventive Dental Science, University of Manitoba, Winnipeg, MB, Canada; ^5^Department of Computer Science, University of Manitoba, Winnipeg, MB, Canada; ^6^Department of Pediatrics and Child Health, University of Manitoba, Winnipeg, MB, Canada

**Keywords:** dental plaque, oral swab, bacteria, fungi, microbiota, machine learning, case-control, artificial intelligence

## Abstract

The human oral cavity harbors one of the most diverse microbial communities with different oral microenvironments allowing the colonization of unique microbial species. This study aimed to determine which of two commonly used sampling sites (dental plaque vs. oral swab) would provide a better prediction model for caries-free vs. severe early childhood caries (S-ECC) using next generation sequencing and machine learning (ML). In this cross-sectional study, a total of 80 children (40 S-ECC and 40 caries-free) < 72 months of age were recruited. Supragingival plaque and oral swab samples were used for the amplicon sequencing of the V4-16S rRNA and ITS1 rRNA genes. The results showed significant differences in alpha and beta diversity between dental plaque and oral swab bacterial and fungal microbiomes. Differential abundance analyses showed that, among others, the cariogenic species *Streptococcus mutans* was enriched in the dental plaque, compared to oral swabs, of children with S-ECC. The fungal species *Candida dubliniensis* and *C. tropicalis* were more abundant in the oral swab samples of children with S-ECC compared to caries-free controls. They were also among the top 20 most important features for the classification of S-ECC vs. caries-free in oral swabs and for the classification of dental plaque vs. oral swab in the S-ECC group. ML approaches revealed the possibility of classifying samples according to both caries status and sampling sites. The tested site of sample collection did not change the predictability of the disease. However, the species considered to be important for the classification of disease in each sampling site were slightly different. Being able to determine the origin of the samples could be very useful during the design of oral microbiome studies. This study provides important insights into the differences between the dental plaque and oral swab bacteriome and mycobiome of children with S-ECC and those caries-free.

## Introduction

The oral cavity harbors one of the most diverse microbial communities within the human body (Stearns et al., 2011). A variety of oral niches (non-shedding tooth surfaces, tongue, cheek, hard and soft palates, and gingival sulcus) provide different levels of oxygen, nutrients, salivary flow, and masticatory forces (Hall et al., 2017). Each of these different microenvironments allow the colonization of unique and adapted microbial communities. Therefore, it is expected that the microbial composition of each oral site differs significantly from each other.

Usually, the oral microbiota exists in a homeostatic balance with the host and contributes to the development of the immune system. However, once this balance is disturbed, some microbial species can overgrow and diseases associated with site-specific microbes such as periodontitis (subgingival microbiota), dental caries (supragingival microbiota), and oral candidiasis (oral mucosal and salivary microbiota) may occur (Lamont et al., 2018; Vila et al., 2020). Therefore, it is important to select the most appropriate site of sampling for the study and/or diagnosis of each oral infectious diseases. Recent studies have shown that the SARS-CoV-2 virus, which causes the coronavirus disease 19 (COVID-19), can be detected in saliva (Fernandes et al., 2020). It has been reported that salivary glands can be important reservoir of the virus (Xu et al., 2020b). Consequently, the presence of high SARS-CoV-2 viral load in saliva could make it a suitable diagnostic tool for COVID-19. Therefore, this also validates the importance of exploring different sampling options for diagnosis of infectious diseases (Fernandes et al., 2020; Sapkota et al., 2020; Xu et al., 2020a).

Since the nineteenth century, it is known that the oral microbes play a crucial role in the development of dental caries (Russell, 2009). However, the establishment of new technologies, such as next generation sequencing (NGS) and machine learning algorithms, has provided a unique opportunity to an enhanced understanding of the role of oral microbes (bacteria, fungi, and viruses) on caries development and progression.

As dental caries continues to be one of the most prevalent chronic diseases among children worldwide, there is a clear need for a deeper understanding of how oral microbial communities and their interactions could impact children’s oral health. The terms early childhood caries (ECC) and severe ECC (S-ECC) were first introduced in the 1990s (Ismail and Sohn, 1999). ECC is described as any caries experience in the primary dentition of children younger than 6 years of age. S-ECC is the severe form of ECC and has an important effect on children’s development and well-being (Pierce et al., 2019; Folayan et al., 2020).

We hypothesized that the microbial (bacterial and fungal) profile of dental plaque significantly differs from that of oral swabs, and because the dental biofilm is in closer contact with the tooth surface, it would provide a better prediction model for caries onset. To test this hypothesis, first we characterized the differences between the dental plaque and oral swab bacterial and fungal microbiota in children with S-ECC and those caries-free. Second, we analyzed which of those commonly used sampling sites (dental plaque and oral swab) would provide a better model for the classification of S-ECC vs. caries-free, using machine learning approaches. Third, we further evaluated whether the observed differences between the microbial profiles of the samples could be used for the differentiation between the sampling sites (dental plaque vs. oral swab) to assist researchers during the design of oral microbiome studies. This is one of the first studies to explore the oral microbiome profiles to classify oral sites.

## Materials and Methods

### Study Population

In this cross-sectional study, eighty children < 72 months of age were recruited between December 2017 and August 2018. Among those, 40 had S-ECC, according to the American Academy of Pediatric Dentistry definition (AAPD, 2020), and 40 were caries-free. Children with S-ECC were recruited at the Misericordia Health Centre (MHC), Winnipeg-MB, Canada, on the day of their full-mouth rehabilitative dental surgery under general anesthesia. Caries-free children were recruited from the community. Caries-free children had a dmft (cumulative score of the number of decayed, missing, or filled primary teeth) index equal to zero and had no incipient lesions. To confirm the caries-free status, a dental examination was performed by R.J.S. at the Children’s Hospital Research Institute of Manitoba by means of visual/tactile examination using artificial light and no radiographs. Inclusion criteria: children less than 72 months of age who were caries-free (dmft = 0) or have been diagnosed with S-ECC (based on the American Academy of Pediatric Dentistry definition). Exclusion criteria: children older than 72 months of age, use of antibiotics, and children who did not satisfy the case definition of S-ECC.

Based on the power analysis published by La Rosa et al. (2012) at 5% significance level, with 40 samples per group and the average number of reads of 50,000 per sample our study would achieve a power > 97%. This study protocol was approved by the University of Manitoba’s Health Research Ethics Board (HREB # HS20961–H2017:250) and by the MHC, Winnipeg, MB, Canada. Written informed consent was provided by the parents or legal caregivers (de Jesus et al., 2020). This work follows the STROBE guidelines checklist for cross-sectional studies (Supplementary Table).

### Sample Collection

Due to the young age of the participants and their inability to spit saliva, oral swab samples were collected with a sterile polyester-tipped applicator (Fisher Scientific) by swabbing the buccal mucosa and anterior floor of the mouth under the tongue. The oral swabs were stored in RNAprotect Reagent (Qiagen, Cat. # 74324, Hilden, Germany) at −80∘C until further analysis. Supragingival plaque samples were collected from all available tooth surfaces with a sterile interdental brush (Agnello et al., 2017; de Jesus et al., 2020). They were dislodged into the RNAprotect Reagent (Qiagen, Cat. # 76506, Hilden, Germany) and stored at −80∘C until further analysis. For simplicity, supragingival plaque samples are referred to as dental plaque.

### DNA Extraction and 16S and ITS1 rRNA Amplicon Sequencing

Total DNA was extracted from 160 samples (80 oral swabs and 80 dental plaque samples) using QIAamp DNA mini kit (Qiagen, Hilden, Germany) following manufacturer‘s protocol. An additional enzymatic digestion step with lysozyme treatment (20 μg/ml lysozyme in a buffer containing 20 mM Tris HCl, pH 8; 1.2% Triton X 100; 2 mM EDTA) was performed before DNA extraction from dental plaque samples.

The total DNA was sent on dry ice to McGill University–Génome Québec Innovation Center (Montreal, Canada) for paired-end Illumina MiSeq PE250 sequencing. The primers 515F, (5′-GTGCCAGCMGCCGCG GTAA-3′) and 806R (5′-GGACTACHVGGGTWTCTAAT-3′), targeting the V4 hypervariable region of the bacterial 16S rRNA gene and the primers ITS1-30 (5′-GTCCCTGCCCTTTGTACACA-3′) and ITS1-217 (5′-TTTCGCTGCGTTCTTCATCG-3′), targeting the Internal Transcribed Spacer 1 (ITS1) of the fungal rRNA gene were used for amplification (Usyk et al., 2017; de Jesus et al., 2020).

### Bioinformatics and Statistical Analysis

The sequences were received as demultiplexed, barcode removed, paired ends fastq files. The quality control analysis was performed with FastqC v0.11.8 (Andrews, 2010). The sequences were then imported and analyzed with QIIME2 2018.11 (Bolyen et al., 2019). The 16S pair-end sequences were quality trimmed, filtered to remove ambiguous and chimeric sequences, and merged using DADA2 implemented in QIIME2, resulting in the amplicon sequence variant (ASV) table (Callahan et al., 2016). The ITS1 pair-end sequences were trimmed using the Q2-ITSxpress QIIME2 plugin prior to the DADA2 step, with default parameters (Rivers et al., 2018). The taxonomic assignment of ASVs was performed using the Human Oral Microbiome Database (HOMD, version 15.1) for bacteria and the UNITE database (version 8.2; QIIME developer release) for fungi at 99% sequence similarity (Dewhirst et al., 2010; Agnello et al., 2017; Abarenkov et al., 2020b; de Jesus et al., 2020). Due to the presence of many fungal ASVs that were assigned only at kingdom level, further fungal ASV curation was performed with the R package LULU (Frøslev et al., 2017). The remaining ASVs assigned as *Fungi* at kingdom level only, or with unidentified phylum were manually assessed using the program BLASTN in NCBI (Zhang et al., 2000). The ASVs with non-fungal BLASTN results were discarded and the remaining were repeatedly assigned to new taxonomic assignments using different UNITE databases threshold levels (Abarenkov et al., 2020a,b,c) and taxonomy classification methods (q2-feature-classifier classify-sklearn and classify-consensus-blast) in QIIME2, as described previously (Martinsen et al., 2021). The data was imported into R using the R package “qiime2R” (version 0.99.13) and additional filtering was performed using “phyloseq” (version 1.30.0) to remove singletons and samples with less than 1,000 reads (McMurdie and Holmes, 2013; Bisanz, 2018; Depner et al., 2020). The ASV counts were then normalized using the cumulative-sum scaling (CSS) approach from the R package “metagenomeSeq” version 1.28.2 (Paulson et al., 2013).

The alpha diversity analyses (within-samples) were performed using the Chao1 and Shannon indices to estimate richness and diversity, respectively, using raw ASV count data from QIIME2 in “phyloseq”. Pairwise comparisons of alpha diversity were done by the paired Wilcoxon signed rank test. Beta diversity measures were calculated on CSS normalized ASV data. This analysis was performed to compare the structure of the bacterial and fungal microbial communities between samples, using the permutational analysis of variance (PERMANOVA) test with 999 permutations in the R package “vegan” (adonis function; version 2.5.6) (Anderson, 2001). It was visualized using principle coordinate analysis (PCoA) with Bray-Curtis dissimilarity index in the R package “ggplot2” (version 3.3.3) (Beals, 1984; Wickham, 2016).

Differentially abundant species were identified using the DESeq2 negative binomial Wald test, controlling the false discovery rate (FDR) for multiple comparison, within “phyloseq” (Love et al., 2014). For this, the raw ASV counts were collapsed to the species level. For comparisons between dental plaque vs. oral swab, a paired DESeq2 analysis was performed. FDR adjusted *P* < 0.05 was considered significant.

### Machine Learning Analysis

Machine learning methods were used to train multivariable classification models to identify the caries status, S-ECC and caries-free. To generate the machine learning models, taxonomic features were used in the form of ASV tables collapsed to species-level. For the classification, we used the workflow provided in “Siamcat,” which provides a machine learning toolbox for metagenome analysis through state-of-the-art machine learning methods (Wirbel et al., 2019, 2021). The data were separately processed for fungi and bacteria and sample-wise relative abundance for the microbiome quantitative profiles was used as input data to maintain the uniformity.

To process the data in “Siamcat,” features with a prevalence of less than five percent across samples were removed and the remaining features were normalized by centered log-ratio (CLR) transformation. The data was then prepared for cross-validation with eightfold and 5 repeats. After this, the models were trained using Lasso, Ridge, Elastic Net (Enet), and RandomForest classification methods in Siamcat, which uses the “mlr” package for machine learning based classification (Bischl et al., 2016). The models’ performance for cross validation was evaluated using the area under the receiver operating characteristic (AUROC) value. To show the importance of the model features, the model feature weights were converted to relative weights and up to the top 20 features were selected, based on their median values, to generate a heatmap using the R package “ggplot2” (Wickham, 2016).

For the machine-learning based classification of plaque and swab samples, a pairwise sample analysis was performed using a boosting conditional logistic regression from R package “clogitboost,” which takes the paired nature of the dental plaque and oral swab samples into account (Shi and Yin, 2015). The model was fitted using component-wise smoothing spline. The caries-free and S-ECC samples were divided into training and test sets using three-quarters of the data for training and the remaining for test in a way that paired samples for plaque and swab should be together in either training or test sets. For the features (species) selection in training dataset, we obtained the *p*-values from the differential abundance analysis described above. The top features selected by the *p*-values were used to train the classification models. Since we have only 30 independent samples in the training set, we considered only top 5, 10, 15, 20, and 25 features to build the model, respectively. The models’ performance was evaluated using AUROC. Each of the trained models were then tested on the test set. The training-test strategy/process was repeated for 30 iterations and the classification performance between caries-free and S-ECC samples were compared by the average of AUROC values from the 30 repeats.

## Results

Eighty children who fit the study criteria were recruited and 160 samples (80 dental plaque and 80 oral swabs) were collected. The Table 1 shows some characteristics of the study participants. Additional information about the participants have been recently published (de Jesus et al., 2020).

**TABLE 1:** Characteristics of study participants*.

	**Caries status**	
	**S-ECC**	**Caries-free**
	**(*N* = 40)**	**(*N* = 40)**
Age (months), mean ± SD	45.6 ± 11.4	46.2 ± 14.2
**Sex, n(%)**		
Female	25 (62.5)	21 (52.5)
Male	15 (37.5)	19 (47.5)

*Other demographics of the study participants have been previously published (de Jesus et al., 2020).

### Bacterial Community Analysis

**FIGURE 1:**
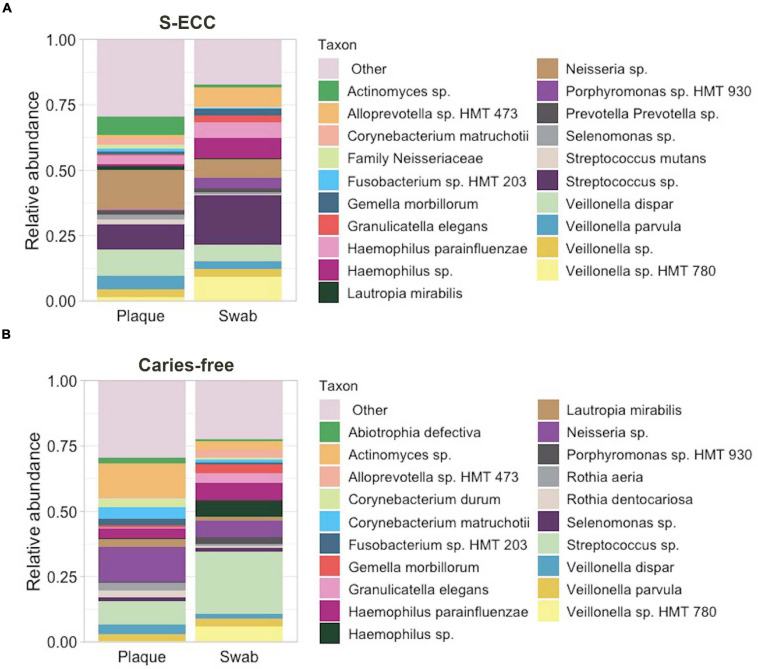
Bacterial taxonomic profiles of dental plaque and oral swab. Relative abundance of the top 20 bacterial taxa in dental plaque and oral swab samples from **(A)** children with S-ECC and **(B)** caries-free children. “Other” indicates the taxa not individually shown. S-ECC, severe early childhood caries.

After filtering out low quality and chimeric sequences, a total of 8,664,777 16S rRNA reads were obtained, with an average number of 54,154.9 reads per sample (160 samples). A total of 5,421 ASVs were assigned to 141 genera and 320 species. Overall, the most abundant phyla were *Firmicutes* (41.08%) and *Proteobacteria* (27.37%). In oral swabs, *Streptococcus* (overall: 21.81%; S-ECC: 19.22%; Caries-free: 24.41%) was the most abundant genus followed by *Veillonella* (overall: 17.03%; S-ECC: 21.65%; Caries-free: 12.40%) and *Haemophilus* (overall: 13.28%; S-ECC: 13.62%; Caries-free: 12.94%). In dental plaque, *Neisseria* (overall: 16.06%, S-ECC: 15.93%; Caries-free: 16.13%), *Veillonella* (overall: 13.66%; S-ECC: 19.54%; Caries-free: 7.73%), and *Streptococcus* (overall: 11.35%; S-ECC: 12.77%; Caries-free: 9.92%) were the most abundant genera. The taxonomic profile of the dental plaque and oral swab samples are shown in Figures 1A,B.

**FIGURE 2:**
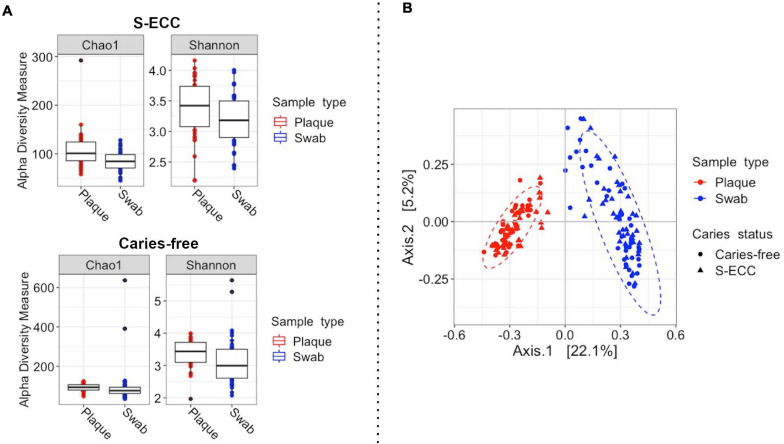
Bacterial diversity of dental plaque and oral swab samples from children with S-ECC and those caries-free. **(A)** For alpha diversity (within-sample) the Shannon and Chao1 diversity and richness measures were calculated according to sample type in both caries-free and S-ECC groups. A significant difference between oral swab and dental plaque alpha diversity and richness was observed in both caries-free and S-ECC groups (*P* < 0.05, paired Wilcoxon test). **(B)** For beta (between-sample) diversity, Bray-Curtis distances were calculated, followed by principal coordinates analysis (PCoA). The plot shows the separation of samples according to sample type (pseudo-F = 40.4, *R*^2^ = 0.2, *P* = 0.001, PERMANOVA accounting for the children’s caries-status). The ellipses represent a 95% confidence level. S-ECC, severe early childhood caries.

Bacterial alpha diversity (within samples) analysis showed a significant difference between oral swab and dental plaque alpha diversity (Shannon index, S-ECC: *P* = 0.0034; Caries-free: *P* = 0.015) and richness (Chao1 index, S-ECC: *P* < 0.001; Caries-free: *P* = 0.025) in both caries-free and S-ECC groups (Figure 2A). Bacterial beta (between-sample) diversity analysis showed a clear separation of samples according to sampling site, oral swab and dental plaque (pseudo-F = 42.71, *R*^2^ = 0.2, *P* = 0.001, PERMANOVA accounting for the children’s S-ECC status and the paired samples; Figure 2B). A significant difference in bacterial community was also observed between the S-ECC and caries-free groups (pseudo-F = 2.85, *R*^2^ = 0.014, *P* = 0.001).

**FIGURE 3:**
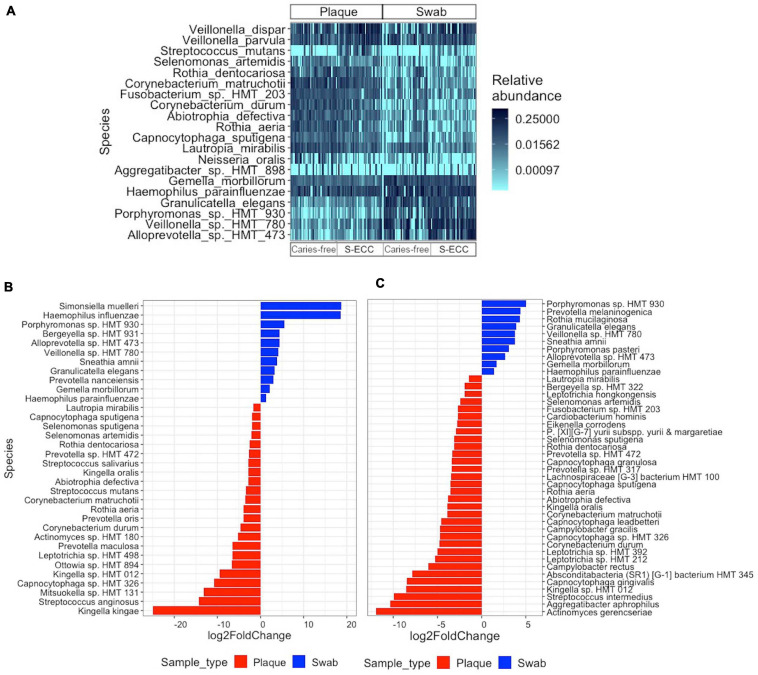
Differential abundance of bacterial species. **(A)** Heatmap showing the relative abundance of the top 20 bacterial species identified in all dental plaque and oral swab samples. **(B,C)** Relative fold change in the abundance of bacterial species in **(B)** samples from children with S-ECC, and **(C)** samples from caries-free children, according to sample type. The differential abundance of the bacterial species was tested using the DESeq2 negative binomial Wald test. **(B,C)** All species listed have an FDR adjusted *P* < 0.05. S-ECC, severe early childhood caries.

Figure 3A shows the relative abundance of the top 20 bacterial species across the subgroups. The differential abundance analysis revealed numerous species that were overabundant in dental plaque or oral swab samples within the S-ECC and caries-free groups (Figures 3B,C, adjusted *P* < 0.05, DESeq2). Interestingly, many species were significantly more abundant in dental plaque or oral swab in both S-ECC and caries-free groups. For instance, *Capnocytophaga* sp. oral taxon 326 (S-ECC: −10.83 log2fold change; Caries-free: −4.72 log2fold), *Kingella* sp. oral taxon 012 (S-ECC: −9.52 log2fold change; Caries-free: −8.57 log2fold change), *Corynebacterium durum* (S-ECC: −4.71 log2fold change; Caries-free: −4.81 log2fold change), *Rothia aeria* (S-ECC: −3.93 log2fold change; Caries-free: −3.56 log2fold change), *Corynebacterium matruchotii* (S-ECC: −3.54 log2fold change; Caries-free: −3.90 log2fold), among others, were more abundant in dental plaque than oral swabs in both caries-free children and those with S-ECC. On the other hand, *Porphyromonas* sp. oral taxon 930 (S-ECC: 5.57 log2fold change; Caries-free: 5.01 log2fold change), *Alloprevotella* sp. oral taxon 473 (S-ECC: 4.35 log2fold change; Caries-free: 2.66 log2fold change*), Veillonella* sp. oral taxon 780 (S-ECC: 4.12 log2fold change; Caries-free: 3.79 log2fold change), *Sneathia amnii* (S-ECC: 3.86 log2fold change; Caries-free: 3.76 log2fold change*) Granulicatella elegans* (S-ECC: 3.26 log2fold change; Caries-free: 3.93 log 2fold change), and *Haemophilus parainfluenzae* (S-ECC: 1.26 log2fold change; Caries-free: 1.39 log2fold change) were more abundant in oral swabs in both caries-free and S-ECC groups. In children with S-ECC, the well-known cariogenic bacterium *Streptococcus mutans* was more abundant in dental plaque samples (−3.45 log2fold change, adjusted *P* < 0.05).

Within the oral swab samples, three species were more abundant in S-ECC compared to caries-free: *Veillonella dispar* (2.09 log2fold change), *Prevotella veroralis* (23.33 log2fold change), and *Neisseria bacilliformis* (24.58 log2fold change, adjusted *P* < 0.05, DESeq2). While *Lautropia mirabilis* (−1.41 log2fold change) was significantly more abundant in caries-free children’s oral swabs (adjusted *P* < 0.05, DESeq2). The differences between the dental plaque microbial composition between children with S-ECC and those caries-free have been previously published (de Jesus et al., 2020). The proportion of bacterial and fungal ASVs assigned to different taxonomic levels are shown in Supplementary Figure 1.

### Fungal Community Analysis

A total of 8,000,067 filtered ITS1 rRNA reads were obtained, with an average number of reads per sample of 50,000.42 (160 samples). The 622 ASVs where assigned to 63 genera and 59 species. After filtering, ten samples had low reads (<1,000) and were removed from the fungal analysis as well as their respective oral swab or dental plaque pairs, resulting in a total sample size of 140. Differential abundance analysis showed that among the top 20 most abundant fungal taxa, within the S-ECC group, *Stereum rugosum* (−29.03 log2fold change), *Fusarium* sp. (−29.14 log2fold change), *Trichoderma* sp. (−24.79 log2fold change), *Candida albicans* (−10.42 log2fold change), *C. dubliniensis* (−6.04 log2fold change) and others were enriched in dental plaque. While *Trichosporon asahii* (23.97 log2fold change), *Malassezia globosa* (20.36 log2fold change), *M. restricta* (14.9 log2fold change) and others were more abundant in oral swabs. Within the caries-free group, the class Agaricomycetes (13.63 log2 fold change) was more abundant in oral swab, while *Blumeria* sp. (−29.94 log2fold change), *Fusarium* sp. (−23.26 log2fold change), *Wallemia tropicalis* (−22.68 log2fold change), *Malassezia restricta* (−16.44 log2fold change) and others were more abundant in dental plaque. Within the oral swab samples, *Candida dubliniensis* (12.92 log2fold change), *Candida tropicalis* (24.99 log2fold change), and *Malassezia restricta* (24.14 log2fold change) were more abundant in children with S-ECC compared to caries-free controls (Table 2, adjusted *P* < 0.05, DESeq2). The results of the differential abundance analysis according to caries status in dental plaque (caries-free vs. S-ECC) have been published previously (de Jesus et al., 2020).

**TABLE 2:** Mean relative abundance of the top 20 most abundant fungal taxa.

	**S-ECC**		**Caries-free**	
**Species**	**Plaque**	**Swab**	**Plaque**	**Swab**
Candida dubliniensis* ^#^	47.76 ± 44.28	13.09 ± 25.16	0.01 ± 0.03	0.00 ± 0.002
Class Agaricomycetes* ^§^	2.52 ± 12.30	11.06 ± 25.85	1.98 ± 6.94	7.21 ± 18.82
Candida albicans*	9.51 ± 24.79	3.59 ± 14.27	5.16 ± 18.28	1.58 ± 6.65
*Blumeria* sp. ^§^	3.1 ± 16.65	0.00 ± 0.00	15.08 ± 30.59	0.00 ± 0.00
Family Thelephoraceae*^ #^	2.165 ± 5.35	0.001 ± 0.01	12.85 ± 26.20	1.14 ± 4.07
Malassezia restricta* ^§^^ #^	0.29 ± 1.03	5.45 ± 18.25	5.55 ± 19.88	0.07 ± 0.38
*Candida tropicalis*^#^	3.90 ± 14.90	2.90 ± 9.77	0.00 ± 0.00	0.01 ± 0.04
Trichosporon asahii* ^§^	0.00 ± 0.00	1.34 ± 8.06	2.99 ± 17.14	0.09 ± 0.56
Ramicandelaber taiwanensi* ^§^	0.52 ± 1.64	0.09 ± 0.52	3.58 ± 7.00	0.05 ± 0.21
*Fusarium* sp.* ^§^	0.58 ± 2.46	0.00 ± 0.00	3.01 ± 17.14	0.00 ± 0.00
*Meyerozyma guilliermondii*^§^	0.10 ± 0.59	0.00 ± 0.00	3.00 ± 17.14	0.00 ± 0.00
*Exophiala radices*	0.00 ± 0.00	0.00 ± 0.00	2.65 ± 15.46	0.00 ± 0.00
*Candida parapsilosis*	0.36 ± 2.16	0.02 ± 0.11	2.204 ± 12.75	0.00 ± 0.00
Malassezia globosa*	0.00 ± 0.00	2.23 ± 13.41	0.03 ± 0.15	0.00 ± 0.00
Order Malasseziales^§^	0.27 ± 1.63	0.00 ± 0.00	2.06 ± 11.99	0.00 ± 0.00
Stereum rugosum*	2.03 ± 12.17	0.00 ± 0.00	0.00 ± 0.00	0.00 ± 0.00
Phylum Rozellomycota^§^	0.17 ± 0.61	0.10 ± 0.58	1.65 ± 8.99	0.04 ± 0.08
Phylum Chytridiomycota^§^	0.26 ± 0.93	0.08 ± 0.34	1.24 ± 5.69	0.37 ± 0.71
Phylum Ascomycota^§^	0.01 ± 0.05	0.03 ± 0.13	0.55 ± 2.19	1.17 ± 5.02
Wallemia tropicalis*^§^	0.01 ± 0.07	0.00 ± 0.001	0.001 ± 0.01	0.00 ± 0.00

*Adjusted P < 0.05 (DESeq2), dental plaque vs. oral swab in children with S-ECC. ^§^ Adjusted P < 0.05 (DESeq2), dental plaque vs. oral swab in caries-free children. ^#^Adjusted P < 0.05 (DESeq2), S-ECC vs. caries-free in oral swab samples. S-ECC, severe early childhood caries.

**FIGURE 4:**
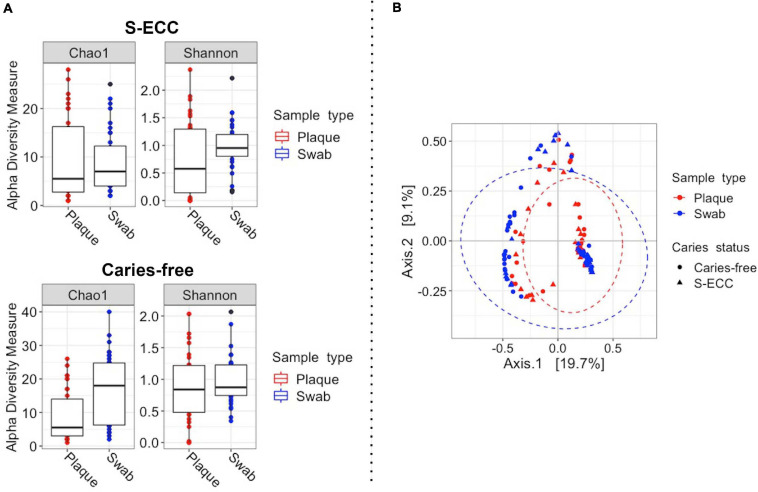
Fungal diversity of dental plaque and oral swab samples. **(A)** For alpha diversity (within-sample) the Shannon and Chao1 diversity and richness measures were calculated according to sample type in both caries-free and S-ECC groups. A significant difference in richness was observed between the sampling sites in the caries-free group (*P* < 0.001, Chao1 index, paired Wilcoxon test). **(B)** For beta (between-sample) diversity, Bray-Curtis distances were calculated, followed by principal coordinates analysis (PCoA, pseudo-F = 11.58, *R*^2^ = 0.04, *P* = 0.001, PERMANOVA). The ellipses represent a 95% confidence level. S-ECC, severe early childhood caries.

The fungal alpha diversity analysis showed a significant difference in Chao 1 diversity (*P* < 0.001, paired Wilcoxon test) in the caries-free group (Figure 4A). Fungal community (β-diversity) analysis also showed a significant difference between dental plaque and oral swab microbiomes (pseudo-F = 5.58, *R*^2^ = 0.04, *P* = 0.001, PERMANOVA; Figure 4B). The fungal communities of samples from caries-free children and those with S-ECC also showed a significant difference (pseudo-F = 4.17, *R*^2^ = 0.03, *P* = 0.001).

### Machine Learning Analysis

We first evaluated the model performance using Lasso, Ridge, Elastic Net (Enet), and RandomForest methods to classify S-ECC vs. caries-free. Overall, the Ridge approach with default parameters provided the best classification accuracy while the other three methods provided similar AUROC values (data not shown). Hence, Ridge was the model of choice for further classification.

**FIGURE 5:**
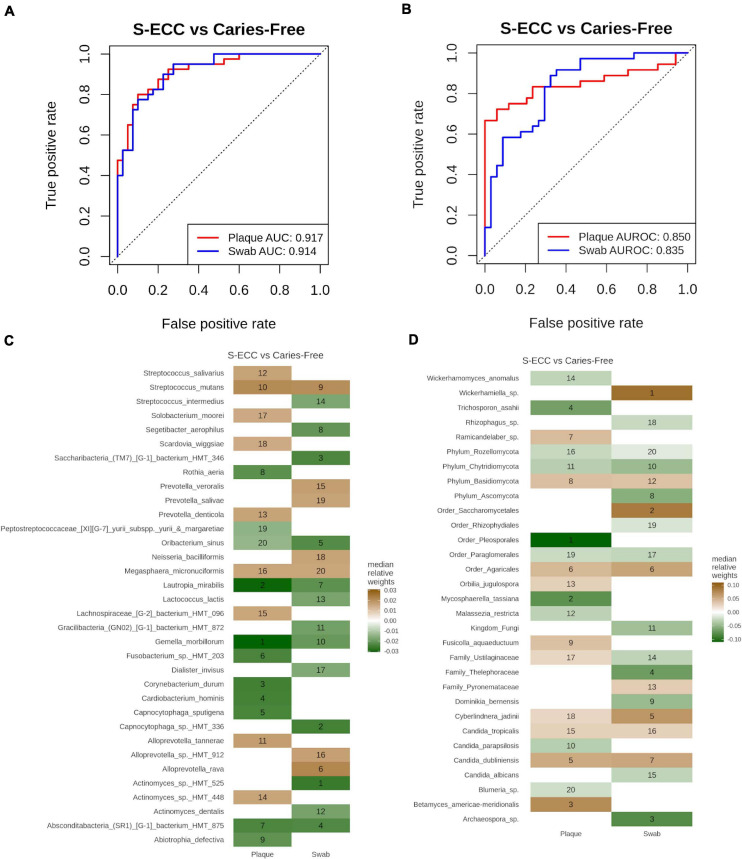
Classification of S-ECC vs. caries-free. **(A,B)** Receiver operating characteristic (ROC) curve representing the cross-validation performance as for the classification of S-ECC and carries-free in **(A)** bacteria and **(B)** fungi using “Ridge” model in Siamcat. The area under the receiver operating characteristic curve (AUROC) represents the sample taken from dental plaques and oral swabs, by red and blue colors, respectively. AUROC values are shown in the bottom-right of the plot. **(C,D)** The relative feature weights used to predict the corresponding model. A maximum of 20 weights in each category were selected to plot on the heatmap and are marked with the ranking of the weights in the heatmap for bacterial **(C)** bacterial and **(D)** fungal taxa. The green color represents the features important in caries-free and brown is for S-ECC.

To evaluate which sampling site, dental plaque or oral swabs, would provide a better classification model for S-ECC vs. caries-free, the samples were grouped according to sampling site. The AUROC values obtained by the Ridge model with bacterial species were 0.92 and 0.91 for dental plaque and oral swab samples, respectively (Figure 5A). While, for fungal taxa, the AUROC values were 0.85 and 0.835, respectively (Figure 5B). The median relative feature weights used to predict the corresponding models and their ranks are shown in Figures 5C,D. Among the most important bacterial features for the S-ECC vs. caries-free classification model are *Gemella morbilorum*, *Lautropia mirabilis*, *Actinomyces* oral taxon 525 and *Capnocytophaga* oral taxon 336. While for fungi, *Mycosphaerella tassiana, Betamyces americae meridionalis, Wickerhamiella* sp. and *Cyberlindnera jadinii* were among the most important discriminatory fungal species.

To evaluate if it is possible to differentiate dental plaque samples from oral swab samples based on their bacterial and fungal profiles, both in caries-free and S-ECC groups, the samples were grouped according to caries status. The AUROC values were compared for the models built based on the top 5, 10, 15, 20, and 25 species selected through differential abundance analysis in the training set. For bacteria, in caries-free samples, the maximum AUROC value was 0.80 using 10 species while for S-ECC, the maximum AUROC value was 0.73 with 25 species. For fungi, the maximum AUROC was obtained by 10 species in caries-free samples and 5 in S-ECC samples (Table 3). The performance of paired analysis for different number of species is summarized in Table 3. It was notable that in site-based classification, in bacteria low number of species provide better classification in caries-free samples. While, for S-ECC samples high number of species are required for improving prediction. For fungi the classification was better with low number of species in both caries-free and S-ECC groups, which might be due to the low alpha diversity in the fungal samples.

**TABLE 3:** Mean AUROC value for plaque vs. swab classification through conditional logistic regression.

	**Bacteria**		**Fungi**	
**Species**	**Caries-free**	**S-ECC**	**Caries-free**	**S-ECC**
5	0.77 ± 0.14	0.67 ± 0.12	0.62 ± 0.17	**0.73 ± 0.15**
10	**0.80 ± 0.13**	0.69 ± 0.15	**0.63 ± 0.18**	0.73 ± 0.16
15	0.73 ± 0.16	0.71 ± 0.17	0.62 ± 0.17	0.69 ± 0.16
20	0.71 ± 0.17	0.72 ± 0.17	0.63 ± 0.19	0.65 ± 0.18
25	0.72 ± 0.16	**0.73 ± 0.17**	0.62 ± 0.17	0.69 ± 0.18

*The species column shows the number of species used in the classification and the mean AUROC values are provided with the standard deviation of 30 iterations of the training-test based prediction. The highest AUROC value of each group is bolded.*

## Discussion

In this study, first we confirmed that the bacterial and fungal community composition of dental plaque differed significantly from that obtained from oral swabs. Second, we investigated, using machine learning approaches, which sampling site would be the most appropriate to differentiate the oral microbial profile of children with S-ECC and those caries-free. Identifying the appropriate type of sample to be used is important to guide future caries association studies. Third, we evaluated whether it could be possible to predict the sampling site (dental plaque vs. oral swab) based on the microbial profile of the samples. Being able to determine the origin of the samples could be useful for the design of future microbiome studies. For instance, if researchers want to collect supragingival plaque, it would be useful to have a way of detecting if during sample collection the supragingival plaque got contaminated with subgingival plaque, as each of those should have unique microbial profiles.

The oral microbiome is considered highly diverse, compared to other body sites. Although dental plaque, saliva and the buccal mucosa are in close contact, they have diverse microbial communities. The Human Microbiome Project (HMP), for instance, compared the diversity of microbes among five major body areas of 242 healthy individuals and showed that supragingival plaque has higher bacterial alpha diversity compared to the oral mucosa, which agrees with the results reported in the present study (The Human Microbiome Project Consortium, 2012). Hall et al. identified a significant difference between the microbial communities of supragingival plaque, saliva, and tongue samples from health subjects, demonstrating the existence of site-specific oral microbiomes (Hall et al., 2017).

Interestingly, while dental plaque showed increased bacterial alpha diversity compared to oral swabs the fungal alpha diversity showed an opposite pattern, with oral swabs displaying increased fungal alpha diversity. The higher fungal diversity observed in the oral swab may be associated with more fungal DNA of transient colonizers from the environment through mouth breathing and food intake (Xu and Dongari-Bagtzoglou, 2015; Diaz and Dongari-Bagtzoglou, 2021). Furthermore, most oral fungi are present at low biomass and may be difficult to detect in oral samples (Diaz and Dongari-Bagtzoglou, 2021). The above factor may explain why the number of observed fungal ASVs was lower than that of bacteria.

Streptococci was the most abundant bacterial genera in oral swabs, similar to what has been previously reported (Caselli et al., 2020). *Neisseria*, *Haemophilus* and *Veillonella*, found to be the most abundant in dental plaque or oral swab samples, have also been reported as highly abundant in different oral sites by previous studies (Huse et al., 2012; Caselli et al., 2020). *Streptococcus, Fusobacterium*, *Gemella*, and *Veillonella* have all been considered core OTUs in different oral sites (Huse et al., 2012; Hall et al., 2017). Here we showed site-specific differences in the abundance of certain species from these genera, with some being significantly more abundant in dental plaque compared to oral swabs or vice-versa. Among children with S-ECC, the known cariogenic bacterium *S. mutans* was significantly enriched in dental plaque samples compared to oral swabs. It also showed to be among the top 10 most important feature for the classification of S-ECC vs. caries-free in both dental plaque and oral swab samples. Other caries associated bacteria such as *Leptotrichia* spp. and *Selenomonas* spp. (Kalpana et al., 2020) were more abundant in dental plaque than oral swab samples from children with S-ECC.

Fungal species from the genera *Candida, Malassezia, Meyerozima*, and *Trichosporon*, were among the most abundant in dental plaque and oral swab, similarly to what has been reported in other studies (Shelburne et al., 2015; Baraniya et al., 2020; Robinson et al., 2020; Diaz and Dongari-Bagtzoglou, 2021). The differential abundance analysis showed a significant difference between *C. dubliniensis* and *C. tropicalis* in the oral swab of caries-free children and children with S-ECC. Those fungal species were also among the top 20 most important features for the classification of S-ECC vs. caries-free in oral swabs. *Candida* spp. are among the most abundant fungal species in the oral cavity and they are associated with different oral diseases (Peters et al., 2017; Diaz et al., 2019). *C. dubliniensis* has only recently been associated with dental caries in children (Al-Ahmad et al., 2016; de Jesus et al., 2020; O’Connell et al., 2020). Here we show that this fungus is not only highly abundant in the dental plaque of children with S-ECC, as previously reported, but it is also enriched in the oral swabs obtained from children with S-ECC compared to those caries-free.

In recent years, machine learning has become a commonly applied approach to early childhood oral health research (Peng et al., 2021). One of the challenges in microbiome data analysis is that the differential analysis methods generally lack the information about predictability. Thus, we used machine learning methods to identify site-specific taxonomic features in dental plaque and oral swabs. The results suggested that both dental plaque and oral swab samples provide a good model for S-ECC vs. caries-free classification. They also suggest that it is possible to differentiate dental plaque from oral swab samples using their microbial profiles. However, site-based classification through fungal species was not optimum in caries-free samples. This could be due to the small number of fungal species that significantly differed in abundance between dental plaque and oral swabs, as observed in the differential abundance analysis.

From our classification results for caries status, it appears that the models using the microbial composition of dental plaque or oral swabs were both able to discriminate between caries-free and S-ECC samples. However, it is important to notice that the species considered to be important for the classification of disease for each sampling site are slightly different. Based on the results from other machine learning models (Lasso, Enet, and RandomForest), we also observed that the choice of the model does not significantly affect the outcome of the analysis (data not shown).

The limitations of this study include, but are not limited to, the lack of information about the socio-economic status of the participants and the convenient sampling used for recruitment, which means that during recruitment the groups were only matched by caries status. As many factors may influence the oral microbial composition, the results of this study may not be generalizable to other populations with different age groups and geographic locations. In this study, an additional enzymatic lysis step was used during DNA extraction from dental plaque samples to disrupt the dental plaque biofilm. Rosenbaum et al. compared the impact of using different DNA extraction methods, including the use of QIAamp DNA Mini Kit (Qiagen) with and without additional enzymatic lysis step, in the oral bacterial (16S rRNA) and fungal (ITS1 rRNA) microbiota. They showed that all tested DNA extraction methods were able to lyse Gram-positive bacterial species. They also reported no significant differences in bacterial and fungal diversity among DNA extraction methods (Rosenbaum et al., 2019). Other studies also found no significant effect of DNA extraction methods in the microbial composition of oral samples (Lim et al., 2017). Therefore, while we do not expect that the additional enzymatic lysis step significantly contributed to the differences observed between the dental plaque and oral swab microbiota, we cannot completely rule out the possible bias associated with the sample preparation on the analyses comparing dental plaque and oral swab microbiomes.

Currently, UNITE is the most commonly used database for taxonomic classification in mycobiome studies of different environments. However, there is an increased concern regarding the lack of taxonomic coverage on the available databases, which creates limitations to studies trying to characterize the human mycobiome (Nilsson, 2016). Here, a high proportion of fungal ASVs (37.14%) could not be classified to a meaningful taxonomic level beyond kingdom. As the reads passed through the quality control process, the observed high number of unclassified ASVs could be a limitation of the database used. Therefore, the construction of a curated ITS database specific for the oral mycobiome, as exists for the oral bacteriome, is urgently needed.

This is a cross-sectional study. Thus, based on our results it is not possible to determine when a significant oral microbial shift from a healthy to a diseased state occurs. Xu et al. performed a longitudinal study where they did a 1-year follow-up of caries-free 3-year-old children (Xu et al., 2018). The authors suggested that prior to any clinical sign of caries, there is a microbial shift that could potentially be used for the diagnosis and prevention of dental caries in young children. Therefore, future longitudinal studies aiming to further characterize the microbial shifts that precede the first clinical signs of dental caries are needed.

In summary, this study characterized the differences in microbial profiles of dental plaque and oral swab samples from children with S-ECC and those caries-free. Importantly, our machine learning results were able to predict the caries-status (S-ECC vs. caries-free) and sampling site (dental plaque vs. oral swab) based on the microbial profile of the samples. In the future, when data from related studies distinguishing oral sampling sites using microbiome profiles are available, we will perform the replication studies to validate our results.

## Data Availability Statement

The datasets presented in this study can be found in online repositories. The names of the repository/repositories and accession number(s) can be found below: https://www.ncbi.nlm.nih.gov/, PRJNA555320, PRJNA714139.

## Ethics Statement

This study protocol was approved by the University of Manitoba’s Health Research Ethics Board (HREB # HS20961–H2017:250) and by the MHC, Winnipeg, MB, Canada. Written informed consent to participate in this study was provided by the participants’ legal guardian/next of kin.

## Author Contributions

VCJ and PC conceived the study. VCJ, MK, PH, and PC contributed to the design, data analysis, interpretation, and writing of the manuscript. VCJ, BAM, and RJS contributed to data acquisition. KD and RJS contributed to the design, data interpretation, and writing of the manuscript. KD, PH, RJS, and PC contributed to funding acquisition. All authors contributed to the article and approved the submitted version.

## Conflict of Interest

The authors declare that the research was conducted in the absence of any commercial or financial relationships that could be construed as a potential conflict of interest.

## Supplementary Material

Supplementary Figure 1 Proportion of ASVs assigned to different taxonomic levels.

## Abbreviations

ASVs: Amplicon Sequence Variants
FDR: False Discovery Rate
HOMD: Human Oral Microbiome database
ITS1: Internal Transcribed Spacer 1
PCoA: Principal Coordinates Analysis
PERMANOVA: Permutational Multivariate Analysis of Variance using distance matrices
S-ECC: Severe Early Childhood Caries.
