# Supplementary figures and images for: Characterization of Supragingival Plaque and Oral Swab Microbiomes in Children With Severe Early Childhood Caries
Source: Front Microbiol. 2021 Jun 25;12:683685. doi: 10.3389/fmicb.2021.683685 (PMC8267818; doi:10.3389/fmicb.2021.683685)

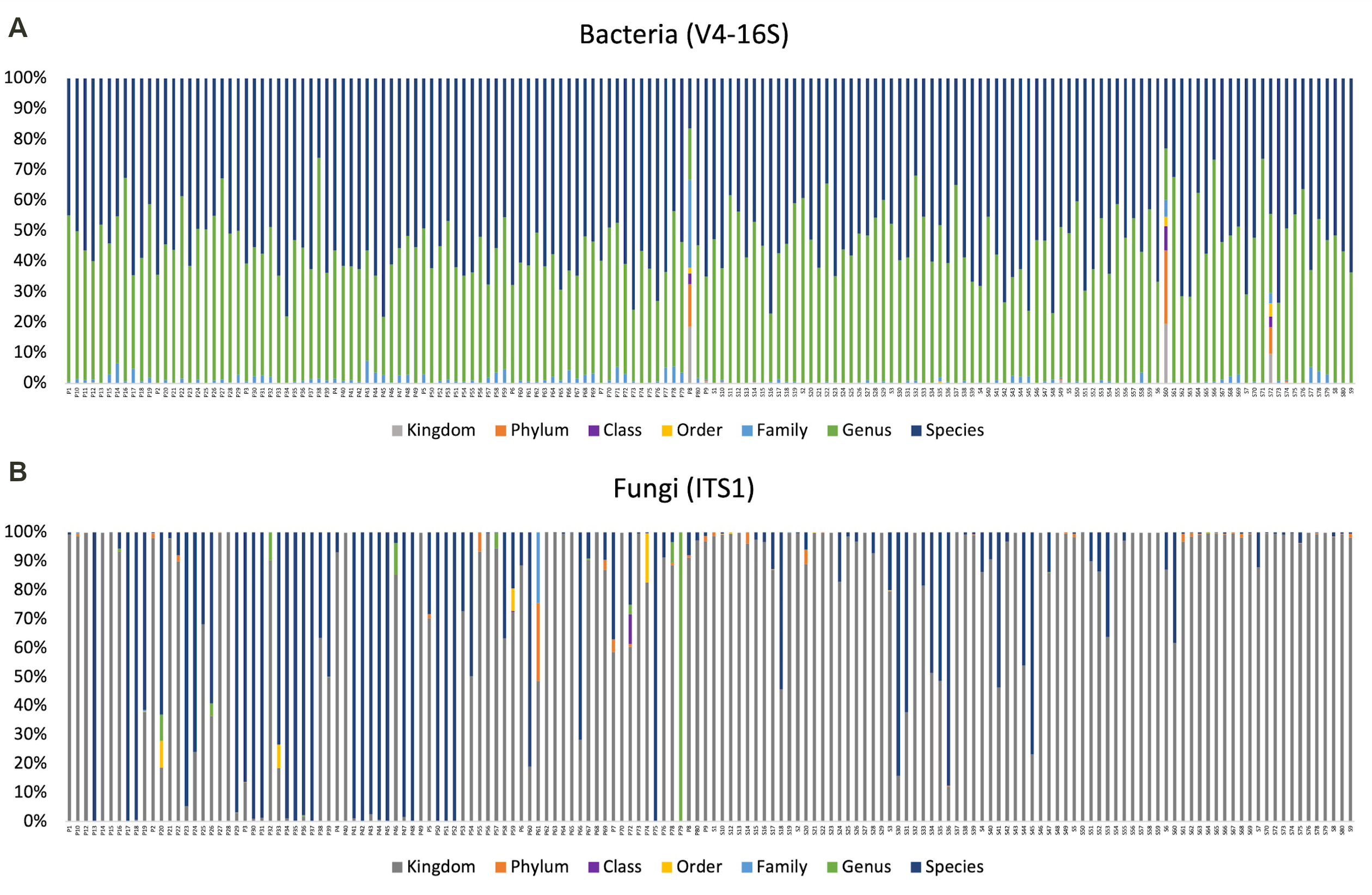

Supplement: Supplementary Figure 1 — Proportion of ASVs assigned to different taxonomic levels. [file Image_1.JPEG]
